# Cloning, characterization of *TaGS3* and identification of allelic variation associated with kernel traits in wheat (*Triticum aestivum* L.)

**DOI:** 10.1186/s12863-019-0800-6

**Published:** 2019-12-18

**Authors:** Jian Yang, Yanjie Zhou, Yu’e Zhang, Weiguo Hu, Qiuhong Wu, Yongxing Chen, Xicheng Wang, Guanghao Guo, Zhiyong Liu, Tingjie Cao, Hong Zhao

**Affiliations:** 1 National Laboratory of Wheat Engineering, Key Laboratory of Wheat Biology and Genetic Breeding in Central Huang-Huai Region, Ministry of Agriculture, Institute of Wheat, Henan Academy of Agricultural Sciences, Zhengzhou, 450002 Henan China; 2 State Key Laboratory of Plant Cell and Chromosome Engineering, Institute of Genetics and Developmental Biology, Chinese Academy of Sciences, Beijing, 100101 China

**Keywords:** *TaGS3*, Kernel traits, Phylogenetic tree, Functional marker

## Abstract

**Background:**

Grain weight is an important yield component. Selection of advanced lines with heavy grains show high grain sink potentials and strong sink activity, which is an increasingly important objective in wheat breeding programs. Rice *OsGS3* has been identified as a major quantitative trait locus for both grain weight and grain size. However, allelic variation of *GS3* has not been characterized previously in hexaploid wheat.

**Results:**

We cloned 2445, 2393, and 2409 bp sequences of the homologs *TaGS3-4A*, *TaGS3-7A*, and *TaGS3-7D* in wheat ‘Changzhi 6406’, a cultivar that shows high grain weight. The *TaGS3* genes each contained five exons and four introns, and encoded a deduced protein of 170, 169, and 169 amino acids, respectively. Phylogenetic analysis of plant GS3 protein sequences revealed *GS3* to be a monocotyledon-specific gene and the GS3 proteins were resolved into three classes. The length of the atypical Gγ domain and the cysteine-rich region was conserved within each class and not conserved between classes. A single-nucleotide polymorphism in the fifth exon (at position 1907) of *TaGS3-7A* leads to an amino acid change (ALA/THR) and showed different frequencies in two pools of Chinese wheat accessions representing extremes in grain weight. Association analysis indicated that the *TaGS3-7A-A* allele was associated with higher grain weight in the natural population. The *TaGS3-7A-A* allele was favoured in global modern wheat cultivars but the allelic frequency varied among different wheat-production regions of China, which indicated that this allele is of potential utility to improve wheat grain weight in certain wheat-production areas of China.

**Conclusions:**

The novel molecular information on wheat *GS3* homologs and the KASP functional marker designed in this study may be useful in marker-assisted breeding for genetic improvement of wheat.

## Background

Wheat (*Triticum aestivum*, AABBDD) provides more than 20% of the calories and 25% of the protein in the human diet (FAOSTAT; http://Faostat.fao.org/site/339/default.aspx and http://www.fao.org/worldfoodsituation/wfs-home/csdben). With the ongoing increase in population size, climate change, and reduction in the available land area, annual gains in crop yields of ~ 2% are required to meet food security [1, 2]. The grain yield of wheat and other cereals is a polygenic trait that is influenced by environmental and genetic interactions at all stages of plant growth [3].

In rice, genes controlling grain weight have been extensively studied [4, 5]. *Grain Size 3* (*GS3*), which encodes a transmembrane protein containing an atypical Gγ domain and a cysteine-rich region, was the first-characterized quantitative trait locus (QTL) that negatively regulates grain length [6, 7] A C–A mutation in the second exon of *GS3* (A allele) is associated with enhanced grain length in *Oryza sativa* but is absent in other *Oryza* species [7]. Pyramiding *GL3.3* (*Grain Length 3.3*) and *GW7* (*Grain Width 7*) enhances yield in an *indica* hybrid rice background and simultaneously improves yield and grain quality [8]. *Grain Size 5* (*GS5*) encodes a putative serine carboxypeptidase and functions as a positive regulator of grain size and yield [9] Analysis of natural variation indicates that mutation of *GS5* is associated with enhanced grain length [10]. *Grain width 5* (*GW5*) encodes a novel nuclear protein of 144 amino acids that is localized to the nucleus and affects grain width [11]. A 1212-bp deletion in rice ‘Nipponbare’ was selected during *japonica* domestication, which functions most likely through influencing the expression levels of *OsGW5*. The GW5 protein is localized to the plasma membrane and can physically interact with and repress the kinase activity of rice GSK2, resulting in accumulation of unphosphorylated OsBZR1 and DLT [12]. A recently reported gene, *GLW7* (*GRAIN LENGTH AND WEIGHT ON CHROMOSOME 7*), also showed a differential expression level in regulating grain weight by allelic variation in the 5′ untranslated region [13]. In addition, *Grain Width 6* (*GW6*) encodes a GNAT-like protein that harbors intrinsic histone acetyltransferase activity, and elevated expression of this gene enhances grain length and weight by enlarging spikelet hulls and accelerating grain filling [14]. *Grain Width 2* (*GW2*) encodes a previously unknown RING-type protein with E3 ubiquitin ligase, which negatively regulates cell division by degrading its substrate(s) by means of the ubiquitin–proteasome pathway [15]. DA2 (orthologous to GW2) is an E3 ubiquitin ligase that physically interacts with DA1 in vitro and in vivo in Arabidopsis [16]. *Grain Length 3* (*GL3*) encodes a protein phosphatase with Kelch-like domains (PPKL) family Ser/Thr phosphatase, which shows epistatic interaction with GS3 and OsGSK3 to modulate brassinosteroid signaling to produce extra-long grains in rice [17–19]. The Kelch domains are essential for *OsPPKL1* (*GL3*) biological function [20]. *DENSE AND ERECT PANICLE 1* (*DEP1*) encodes a highly cysteine-rich G protein gamma subunit protein. Gain-of-function mutation of *DEP1* enhances meristematic activity, resulting in reduction in the length of the inflorescence internodes, an increased number of grains per panicle, and a consequent increase in grain yield [21].

In wheat, the large genome size and complex genome composition renders map-based cloning of functional genes difficult, especially for complex traits. However, the close relationship between wheat and rice provides the opportunity for researchers to clone homologous functional genes with a common ancestor, in particular for development-related traits [22–24]. On the basis of this theory, a number of grain weight-related genes have been cloned and characterized in natural populations and/or bi-parental populations. Orthologs of *TaGW2* have been widely characterized in wheat. Two single-nucleotide polymorphisms (SNPs) forming a Hap-6A-A haplotype for *TaGW2-6A* are associated with broader grains and higher 1000-grain weight [25]. An additional haplotype, Hap-6B-1 for *TaGW2-6B*, shows stronger effects on grain weight than *TaGW2-6A* and this was further validated by gene-editing mutant analysis [26, 27]. A number of reverse genetic studies have shown that *TaGW2* has a negative influence on grain size in hexaploid and tetraploid wheat [28, 29]. Homologs of *GW5* also exert many effects on grain weight and grain size; the haplotype TaGS5-3A-T, based on the SNP T/G in the sixth exon, is significantly correlated with larger grain size, higher 1000-kernel weight, and lower plant height, spike length, and internode length below the spike, which is under strong positive selection in Chinese modern wheat breeding [2, 30]. *TaGW6*, an ortholog of rice *GW6*, encodes a novel indole-3-acetic acid-glucose hydrolase. Variation among wheat homologs of TGW6 on chromosome arms 3AL and 4AL individually explain ~ 17% of TGW variation, and the low-expression alleles are associated with low auxin content and high TGW [31, 32]. *TaGW7* is a homolog of a gene encoding a TONNEAU1-recruiting motif protein, which is mainly expressed in young spike tissues, was shown to negatively regulate grain weight and grain width using CRISPR-Cas9 gene editing analysis. This gene regulates seed size probably effects genes through cell division pathways [33, 34]. Notably, a number of grain trait-related genes (e.g., *TaGW2*, *TaGL3*, and *TaGW8*) were mapped in the QTL region that is likely to harbpr the causative genes [35–37].

*TaGS3-1D* has been cloned in hexaploid wheat and a 40-bp indel predicted to generate a new isoform that is associated with grain weight [38]. However, the hexaploid wheat genome consists of three subgenomes, of which the D genome generally shows the lowest genetic diversity [39, 40]. Therefore, we were interested in isolating the two homologous *TaGS3* genes in the A and B subgenomes and determining the functional variation for potential use in wheat improvement. For this purpose, we cloned the *TaGS3* genes, investigated the evolution of *GS3* genes in plants, designed genetic markers for *TaGS3*, validated its effects in a natural population, and evaluated its distribution in major wheat-production areas of China.

## Results

### Isolation of *TaGS3* in wheat

**Fig. 1:**
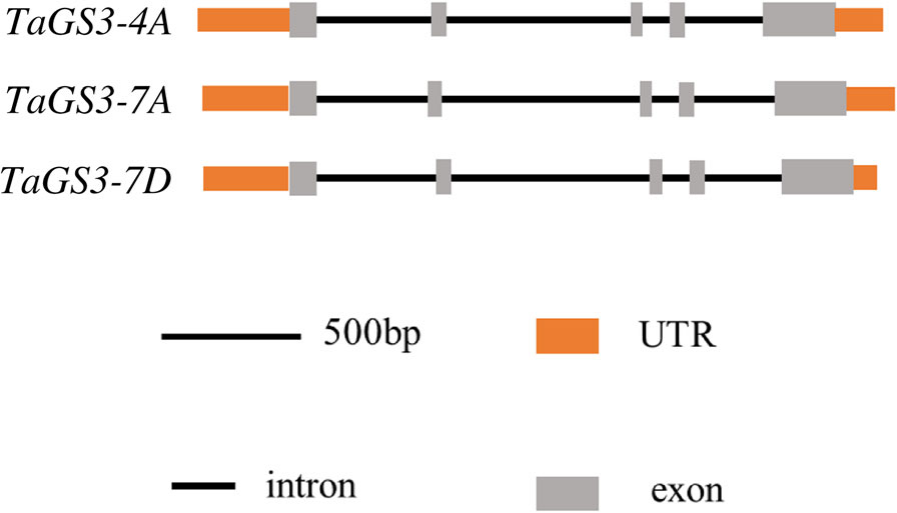
Predicted exon–intron structure of the three *TaGS3* homologues in Changzhi6406

In wheat, three copies of *TaGS3* on chromosome arms 4AL (*TraesCS4A02G474000*), 7AS (*TraesCS7A02G017700*), and 7DS (*TraesCS7D02G015000*) were searched in the Chinese Spring RefSeq v1.0 genome database. Primers were designed based on specific regions for each sequence (Table 1). The primers amplified PCR products 2445, 2393, and 2409 bp in length for *TaGS3-4A* (GenBank accession: KY888174), *TaGS3-7A* (GenBank accession: KY888186), and *TaGS3-7D* (GenBank accession: KY888197) in wheat accession ‘Changzhi 6406’ (a cultivar that exhibits high grain weight). The lengths of the predicted coding sequences of the three genes were 513, 510, and 510 bp, encoding putative proteins of 170, 169, and 169 amino acids, respectively. *TaGS3-4A*, *TaGS3-7A*, and *TaGS3-7D* showed similarity in exon–intron structure to that of *OsGS3*, which consisted of five exons and four introns (Fig. 1). The degree of similarity between the coding region of *OsGS3* and that of *TaGS3-4A*, *TaGS3-7A*, and *TaGS3-7D* was 45.92, 43.94, and 44.87%, respectively. In parallel, the similarity of the deduced amino acid sequences was 45.26, 44.83, and 42.24%, respectively.

**Table 1:** Primers used in this study

Primer	Sequence (5′-3′)	explanation	predicted PCR product Size
TaGS3-4A-F	CGATCCTTCTCTGCGGCAAG	primers for amplifying *TaGS3-4A*	2435 bp
TaGS3-4A-R	CCTACAGACCGACGACTTCCTG		
TaGS3-7A-F	CGACTTCCTGTCTCCTTCCGG	primers for amplifying *TaGS3-7A*	2389 bp
TaGS3-7A-R	TCATGCCCGTCAAAAACACCAG		
TaGS3-7D-F	GACGACTTCCTGTCTCCTACTTCC	primers for amplifying *TaGS3-7D*	2409 bp
TaGS3-7D-R	TCATGCCCGTCAAAAACACCAG		
TaGS3-7A-1907-F1	FAM-GCGCCGGTTGCTCCTCATCTTGCG	KASP marker for distinguishing A/G allele	
TaGS3-7A-1907-F2	HEX-GCGCCGGTTGCTCCTCATCTTGCA		
TaGS3-7A-1907-CR	AGGGACGCCRCCGCAGCACACGGT

### Phylogenetic analysis of GS3 in plants

**Fig. 2:**
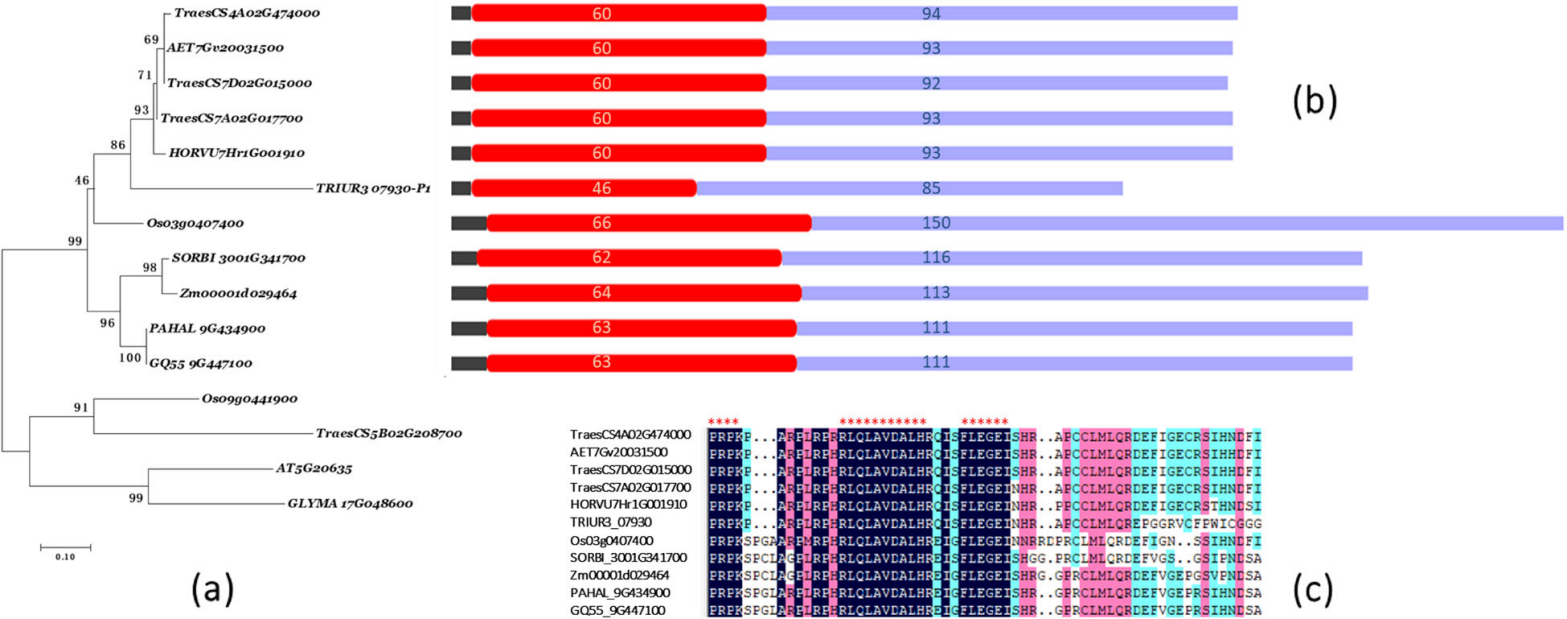
**A** Phylogenetic analysis of *GS3* orthologous in plants using deduced amino acid sequences. **B** Domain structure of GS3 orthologous in corresponding to left phylogenetic tree. Red solid box represented the conserved atypical Gγ domain and light purple represented cysteine-rich region. The number represented the length of each domain/region. c) Multiple alignment of GS3 homologous sequences. Conserved amino acids are indicated by red asterisk. *TraesCS4A02G474000* (*Triticum aestivum*), *TraesCS7A02G017700* (*Triticum aestivum*), *TraesCS7D02G015000* (*Triticum aestivum*), *AET7Gv20031500* (*Aegilops tauschii*), *HORVU7Hr1G001910* (*Hordeum vulgare*), *TRIUR3_07930* (*Triticum urartu*), *Os03g0407400* (*GS3*, *Oryza sativa Japonica Group*), *SORBI_3001G341700* (*Sorghum bicolor*), *Zm00001d029464* (*Zea mays*), *PAHAL_9G434900* (*Panicum hallii FIL2*), *GQ55_9G447100* (*Panicum hallii HAL2*), *Os09g0441900* (*DEP1*, *Oryza sativa Japonica Group*), *TraesCS5B02G208700* (*Triticum aestivum*), *GLYMA_17G048600* (*Glycine max*), *AT5G20635* (*Arabidopsis thaliana*)

A phylogenetic tree was reconstructed for 14 deduced amino sequences for GS3 homologs from *Aegilops tauschii*, *Hordeum vulgare*, *Panicum hallii*, *Setaria italica*, *Triticum aestivum*, *Zea mays*, *Triticum urartu*, *Sorghum bicolor*, *Glycine max*, and *Arabidopsis thaliana*. A reported atypical Gγ domain gene, *DEP1*, was used as the outgroup. In the phylogenetic tree the sequences were resolved into two main groups, which comprised the GS3 orthologs and DEP1 orthologs (Fig. 2a). The GS3 orthologous group consisted of ten sequences that were all derived from monocotyledons, whereas the DEP1-orthologous group comprised sequences derived from monocotyledons and dicotyledons.

In the GS3 orthologs group, the relationships among the genes were congruent with the species phylogeny, except that the relationship between TaGS3-7A in wheat subgenome A and TuGS-7A in *Triticum urartu* was more distant than that with GS3 of *Hordeum vulgare*. The similarity of the conserved domain among the sequences was 85.14% overall and the sequences were of similar length (60–66 amino acids) except for that of *T. urartu* (46), whereas the length of the cysteine-rich region was variable and was divided into three classes. Class I consisted of Triticeae species with a conserved length of 92–94 amino acids (85 in *Triticum urartu* was an exception); Class II included *Oryza* species of which the longest sequence was 150 amino acids; and Class III consisted of Panicoideae species with a conserved length of 111–116 amino acids. Comparison of the atypical Gγ subunit conserved domain among the GS3 ortholog sequences revealed that all sequences contained an identical amino acids fragment “PRP--RLQLAVDALHR--FLEGEI” (“--” represents a non-conserved region and the number of “-” does not reflect the number of amino acids).

### Polymorphism of *TaGS3-4A* and *TaGS3-7A* among wheat accessions

Sequences for *TaGS3-4A* and *TaGS3-7A* from ten wheat accessions were obtained. To avoid possible false variation generated by sequencing error, an advanced analysis for detection of real variants resulted in a minor allele frequency > 0.1 (real SNPs must be detected at least twice in ten sequences). In total 17 and 18 variations for *TaGS3-4A* and *TaGS3-7A* was detected by multiple alignment, respectively. There were 3, 1, 6, 0, 3, 0, 0, 0, 0, 2, and 2 variations located in the 5′ upstream region, first exon, first intron, second exon, second intron, third exon, third intron, fourth exon, fourth intron, fifth exon, and the 3′ downstream region for *TaGS3-4A*, respectively. In parallel, 4, 0, 2, 0, 4, 0, 0, 1, 3, 3, and 1 variable sites were detected in the corresponding regions for *TaGS3-7A* (Tables 2 and 3). Among them, both three SNPs causing changes causing amino acid change were determined for each of *TaGS3-4A* and *TaGS3-7A*, which were located in the first exon (1) and fifth exon (2), and in the fourth exon (1) and fifth exon (2), respectively. The variant (GC/AT) at position 70 for *TaGS3-4A* was located in an atypical Gγ subunit, of which the GC allele was harbored by 80 and 60% of accessions grouped into heavy and light grain weight pools, respectively, which indicated this locus was not associated with grain weight. In addition, the frequency of allele A at position 1907 (amino change: ALA/THR) was 0.6 in the heavy grain weight accessions compared with zero in the light grain weight accessions. Therefore, this allele was considered more likely to be associated with grain weight in wheat.

**Table 2:** Allelic variation of *TaGS3-4A*. The position was accounted from ATG (1)

position	location	Changzhi 6406	Sankecun	Shannong 7859	Kangdingxiaomai	Xingyi 4	Youmangbaifu	Dongnong 101	Gansu 96	Fuzhuang 30	Bima 4	amino acid change
− 306	5’UTR	T	C	C	T	T	T	C	T	T	T	–
−129	5’UTR	TG	CG	TG	TG	TG	TG	CA	CG	TG	TG	–
−53	5’UTR	G	A	G	G	G	G	A	A	G	G	–
70	1st exon	GC	AT	GC	GC	GC	GC	AT	AT	GC	GC	A/M
200	1st intron	G	G	G	C	G	G	G	G	C	C	–
250	1st intron	T	T	T	–	T	T	T	T	–	–	–
331	1st intron	T	A	T	T	T	T	A	A	T	T	–
354	1st intron	ACT	G	ACTG	ACTG	G	ACTACTG	G	G	G	G	–
378	1st intron	A	G	A	A	A	A	G	G	A	A	–
397	1st intron	T	T	T	T	C	T	C	T	T	T	–
633	2nd intron	C	T	T	T	C	C	T	T	T	T	–
712	2nd intron	A	T	T	A	A	A	T	T	A	A	–
1140	2nd intron	T	T	T	T	C	T	T	C	T	T	–
1747	5th exon	T	C	T	C	T	T	T	T	T	T	L/P
1897	5th exon	C	C	T	C	T	T	C	C	C	C	A/V
1976	3’UTR	C	C	G	C	G	G	C	C	C	C	–
2004	3’UTR	C	G	C	C	C	C	G	G	C	C	–

**Table 3:** Allelic variation of *TaGS3-7A*. The position was accounted from ATG (1)

position	location	Changzhi 6406	Sankecun	Shannong 7859	Kangdingxiaomai	Xingyi 4	Youmangbaifu	Dongnong 101	Gansu 96	Fuzhuang 30	Bima 4	amino acid change
− 285	5’UTR	T	T	T	C	C	C	T	C	C	C	–
−244	5’UTR	(CCG)_6_	(CCG)_6_	(CCG)_6_	(CCG)_3_	(CCG)_5_	(CCG)_6_	(CCG)_6_	(CCG)_3_	(CCG)_3_	(CCG)_3_	–
− 210	5’UTR	–	–	–	C	–	G	C	C	C	C	–
− 159	5’UTR	C	C	C	T	T	T	T	T	T	T	–
417	1st intron	GCTAGC.....TAGTAGCT	GCTAGC.....TAGTAGCT	GCTAGC.....TAGTAGCT	GCTAGCTACTGTAGTAGCT	GCTAGC.....TAGTAGCT	GCTAGC.....TAGTAGCT	GCTAGC.....TAGTAGCT	GCTAGCTACTGTAGTAGCT	GCTAGCTACTGTAGTAGCT	GCTAGCTACTGTAGTAGCT	–
479	1st intron	T	T	T	–	–	–	–	–	–	–	–
582	2nd intron	C	C	C	T	T	T	T	T	T	T	–
785	2nd intron	G	G	G	C	G	G	G	C	C	C	–
1022	2nd intron	C	C	C	T	T	T	T	T	T	T	–
1117	2nd intron	T	T	T	C	T	T	T	T	C	C	–
1435	4th exon	T	T	C	T	T	T	T	C	T	T	I/T
1515	4th intron	G	G	G	A	G	G	G	A	A	A	–
1523	4th intron	A	A	A	G	A	A	A	A	G	G	–
1543	4th intron	A	A	A	G	A	A	A	A	G	G	–
1814	5th exon	G	G	G	C	G	G	G	C	C	C	G/R
1907	5th exon	A	A	A	G	G	G	G	G	G	G	T/A
1948	5th exon	C	C	C	T	C	C	C	T	T	T	–
2000	3’UTR	TT	CT	TT	AT	CT	TT	TT	A	AT	AT	–

### Molecular marker design and validation

**Fig. 3:**
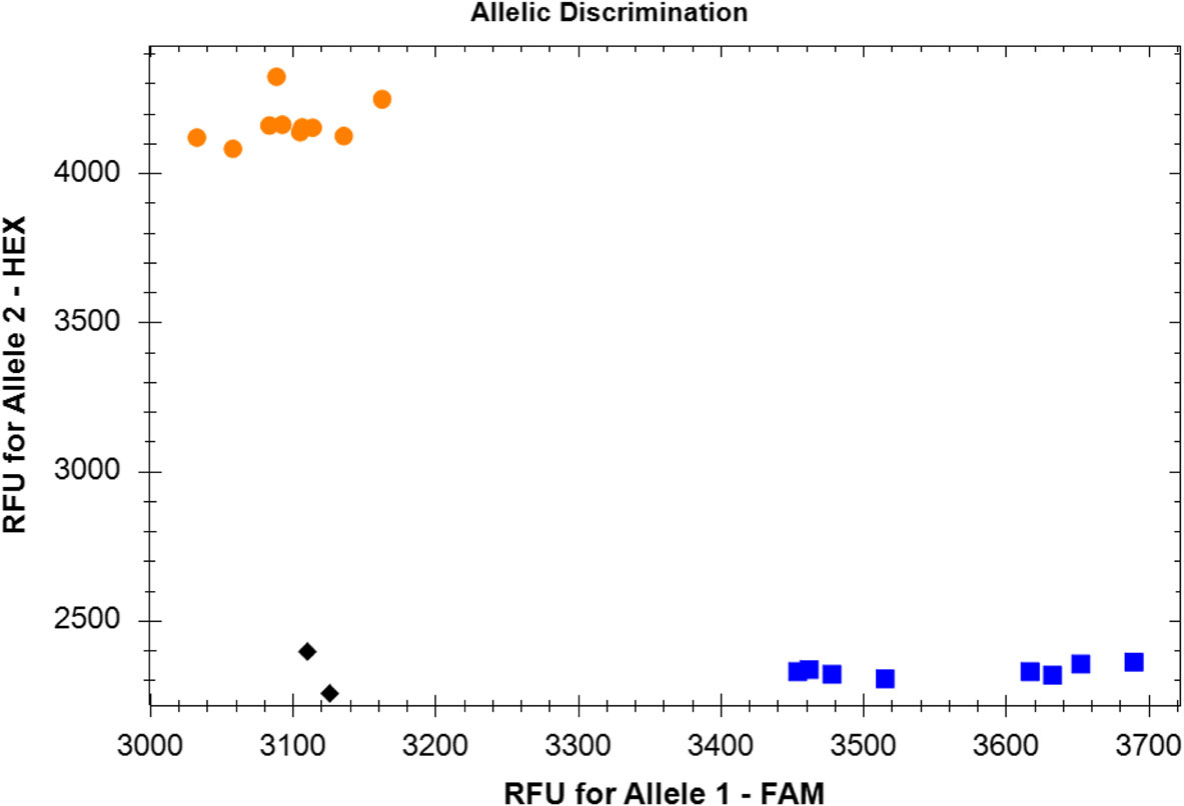
Scatter plots for KASP assays for *TaGS3-7A* showing clustering of varieties on the X- (FAM) and Y- (HEX) axes. Varieties colored blue have the FAM-type allele, for which the clustered samples carried *TaGS3-7A-G* allele showed small grain weight value and grain size; varieties colored orange have the HEX-type allele, the clustered samples carried *TaGS3-7A-A* allele showed larger grain weight value and grain size; black dots represent the NTC (no template control)

Based on the results of SNP mining, a Kompetitive allele-specific PCR (KASP) marker was designed for the SNP at position 1907 (A/G). The calls for the *TaGS3-7A-A* allele of the potential light grain weight group were clustered along with the *x*-axis, whereas the calls of the *TaGS3-7A-G* allele of the potential heavy grain weight group were clustered along with the *y*-axis (Fig. 3). Thus, the clustering results clearly distinguished the two alleles, which was used to validate its effects on grain weight in the natural population.

The association between genotypes and grain traits was analyzed based on a mini-core collection (MCC) of 224 wheat accessions for this marker (Additional file 1: Table S1). On the basis of the SNP calls using the KASP marker, significant differences for kernel width, kernel weight, and kernel thickness was observed for the *TaGS3-7A-G* and *TaGS3-7A-A* alleles, whereas no significant difference for kernel length was observed (Table 4). In addition, the allele *TaGS3-7A-A* with superior traits with a rare frequency of 13.107%, which was significantly less than that for *TaGS3-7A-G*. However, the frequency of *TaGS3-7A-A* in cultivars (18.085%) was significantly higher than that in landraces (0.980%).

**Table 4:** Association analysis of kernel traits between *TaGS3-7A-G* and *TaGS3-7A-A* genotypes. note: * *p*<0.05, ** *p*<0.01

Trait	Allele	N	Mean	Sig
TKW_E1	*TaGS3-7A-G*	116	36.40 ± 0.69	0.010**
	*TaGS3-7A-A*	20	40.96 ± 1.28	
TKW_E2	*TaGS3-7A-G*	140	32.76 ± 0.61	0.075
	*TaGS3-7A-A*	21	35.77 ± 1.44	
TKW_E3	*TaGS3-7A-G*	145	34.58 ± 0.61	0.004**
	*TaGS3-7A-A*	24	39.11 ± 1.22	
GL_E1	*TaGS3-7A-G*	147	0.639 ± 0.005	0.27
	*TaGS3-7A-A*	24	0.654 ± 0.013	
GL_E2	*TaGS3-7A-G*	143	0.643 ± 0.005	0.089
	*TaGS3-7A-A*	22	0.665 ± 0.011	
GL_E3	*TaGS3-7A-G*	145	0.668 ± 0.005	0.134
	*TaGS3-7A-A*	24	0.686 ± 0.010	
GW_E1	*TaGS3-7A-G*	147	0.303 ± 0.003	0.013*
	*TaGS3-7A-A*	24	0.321 ± 0.006	
GW_E2	*TaGS3-7A-G*	143	0.301 ± 0.002	0.007**
	*TaGS3-7A-A*	22	0.316 ± 0.005	
GW_E3	*TaGS3-7A-G*	145	0.306 ± 0.002	0.020*
	*TaGS3-7A-A*	24	0.318 ± 0.006	
GT_E1	*TaGS3-7A-G*	147	0.267 ± 0.002	0.010**
	*TaGS3-7A-A*	24	0.281 ± 0.004	
GT_E2	*TaGS3-7A-G*	143	0.278 ± 0.002	0.005**
	*TaGS3-7A-A*	22	0.292 ± 0.004	
GT_E3	*TaGS3-7A-G*	145	0.289 ± 0.002	0.083
	*TaGS3-7A-A*	24	0.298 ± 0.004	
GL/GW_E1	*TaGS3-7A-G*	147	2.119 ± 0.019	0.221
	*TaGS3-7A-A*	24	2.053 ± 0.059	
GL/GW_E2	*TaGS3-7A-G*	143	2.146 ± 0.016	0.456
	*TaGS3-7A-A*	22	2.113 ± 0.044	
GL/GW_E3	*TaGS3-7A-G*	145	2.190 ± 0.016	0.784
	*TaGS3-7A-A*	24	2.178 ± 0.053	

### Distribution of the *TaGS3-7A-A* allele in the major wheat-production areas of China

In total, 238 wheat cultivars grown in the main wheat-production areas of China were analyzed to determine the frequency of the *TaGS3-7A-A* allele (Additional file 1: Table S2). The *TaGS3-7A-A* allele was detected in 58.4% of all tested cultivars. Significant differences in the frequency of *TaGS3-7A-A* were observed among the five province areas. The *TaGS3-7A-A* allelic frequency exceeded 50% in all provinces except Henan (i.e., 92.0% in Sichuan, 62.5% in Shandong, 60% in Hebei, and 52.6% in Shaanxi). The *TaGS3-7A-A* allele was detected in 50% of the cultivars in Shaanxi province. However, a significantly lower frequency of *TaGS3-7A-A* was detected among cultivars from Henan (28.23%) compared with the other provinces.

## Discussion

### Structure and evolution of GS3

A number of genes that encode G proteins in rice have been cloned and show a variety of functions in the regulation of organ development [10, 21]. *GS3*, which encodes heterotrimeric G proteins that contain an atypical Gγ domain, is a major QTL for grain size [6, 10]. However, the evolutionary history of *GS3* in plants has not been examined previously. In the present study, we performed a phylogenetic analysis of protein sequences using the rice GS3 sequence as a query. There was no GS3 homologs in dicotyledon plant, indicated that GS3 homologs was a monocotyledon-specific gene family. We also determined that the wheat genome contains three GS3 orthologous genes on chromosome arms 7AS, 7DS, and 4AL based on the released ‘Chinese Spring’ reference genome sequence [22]. The molecular evolution of GS3 was consistent with their species evolution relationship. However, the phylogenetic relationship between *TaGS3-7A* and *TuGS3-7A* was more distant than that between *TaGS3-7A* and other Triticeae species, which probably because *TuGS3-7A* was located on 4AL/7BS translocation that is occurred at polyploidizing progress during diploid wheat to polyploid wheat [41–44].

All *GS3* orthologous genes showed a conserved atypical Gγ domain with high similarity, whereas the length of the cysteine-rich region was variable. In particular, the length of the cysteine-rich region in Triticeae species was ~ 60% and ~ 80% the length of that in rice and Panicoideae species, respectively. Although the conserved domain usually determines the function of genes universally, the length of the C-terminal tail may affect degradation of the GS3 protein in rice [45]. Therefore, evolution of *GS3* has led to differences in the length of GS3 in monocotyledons and likely fine-tune of the protein function.

### Polymorphism of *GS3* and its association with grain weight

In the present study, the isolated wheat *TaGS3-4A*, *TaGS3-7A*, and *TaGS3-7D* genes were predicted to contain five exons and four introns, which are identical to *GS3* genes in rice. A large number of variations for *TaGS3-7A* and *TaGS3-4A* were detected. Only one variant located in the GS3 Gγ-subunit was detected in *TaGS3-4A* and this variant seemed to be not associated with grain weight in the different phenotype pools. Nevertheless, the non-conserved domain of GS3 is involved in positive regulation of grain length in rice [46]. Additional polymorphic loci motifs of (AT) n in the fourth intron and (TCC) n in the fifth exon of GS3 were mainly associated with medium to short grains among Chinese rice accessions because 13 accessions harboring the TGC mutation produced long grains, which suggested that the C-A mutation did not completely explain the accessions with long or short grains in Chinese rice germplasm. In maize, two polymorphisms in the fifth exon (not located within the conserved region) and the promoter region are significantly associated with kernel length and 1000-kernel weight In similarity, we also found that variations in the conserved domain region was not associated with grain weight in consideration of allelic frequency between two pools with different phenotype. However, an additional SNP located in the cysteine-rich region that showed a difference in frequencyand thus applied for marker development. This variation probably effect Gβγ dimers from autoinhibition through alternation of the structure of cysteine-rich region [45, 47]. The cysteine-rich region plays a variety of roles in exercise its function. GAST1-like proteins are involved in redox reactions via their cysteine-rich domain and GASA4 mutated by replacement of conserved cysteines with alanines lost its redox activity and the capability to promote gibberellin responses [48]. In addition, the C-terminal cysteine-rich region is sufficient for rice *OsDep1* to confer cadmium tolerance to yeast cells [49]. Collectively, the afore-mentioned findings suggest that variation in the non-conserved domains of GS3 may also affect the phenotype.

In previous studies, one QTL for grain weight was indicated to be associated with *TaGS3-7D* in wheat and all variation between parental lines was contained in the second intron. However, subgenomes A and B show broader genetic diversity compared with that of D subgenome in hexaploid wheat, leading to assessment of the sequence diversity of *TaGS3* and development of a functional marker for *TaGS3* for use in breeding [39, 50]. However, we observed that none of the reported major QTLs for grain weight/size were located in the terminal region of chromosome arm 7AS in the vicinity of *TaGS3-7A*, which was probably because cultivars often carry a weak allelic variation of functional genes, which could provide balance for maintaining the enhancement of grain weight and plant fitness [45, 51, 52]. However, variations with weak effects on phenotype was usually behind high threshold in QTL mapping. Therefore, application of natural accessions with extreme phenotype as a sampling was an alternatively method for detection of functional allelic variation in wheat [31, 53, 54].

**Fig. 4:**
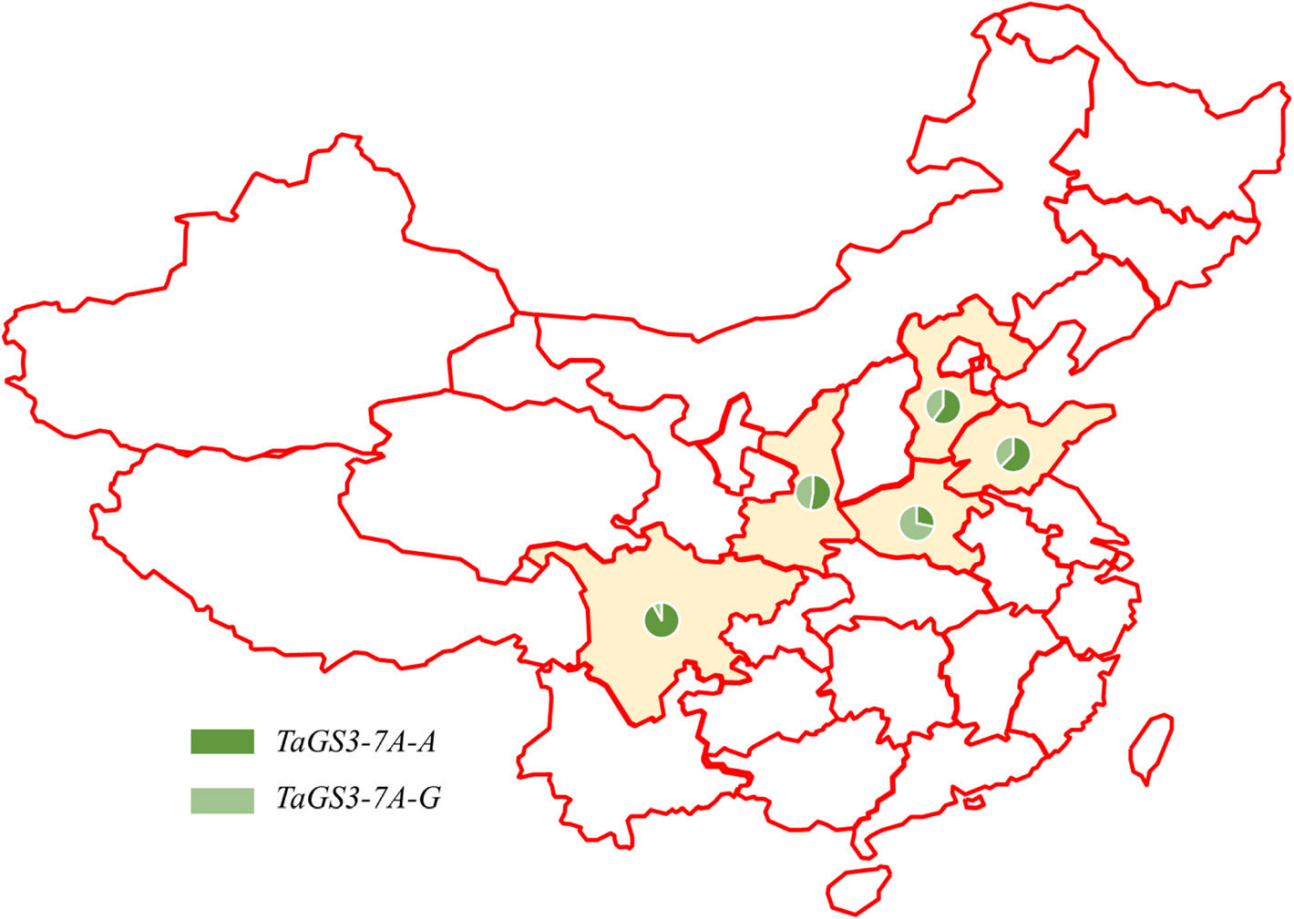
Distribution of the *TaGS3-7A-A* and *TaGS3-7A-G* allele in the main wheat production areas of China

In particular, modern cultivars grown in the main wheat-production areas of China showed a different allele frequency of *TaGS3-7A-A* and exceeded more than 50% of cultivars in most provinces (Fig. 4). The highest frequency of *TaGS3-7A-A* and *TaGS5-3A-T* alleles were detected in southwestern China, which indicated that the alleles were probably strongly selected in this region [2]. In addition, the lowest frequency of the *TaGS3-7A-A* allele was observed in Henan province, which suggested that there remains potential for improvement of grain weight in some wheat-production areas by utilization of the *TaGS3-7A-A* allele.

Cloning of grain weight-related genes and characterization of allelic variation in wheat are essential for molecular breeding. In addition, the functional marker for *TaGS3-7A* developed in the present study is potentially useful for wheat yield improvement.

## Conclusions

In the present study, we cloned homologs of *TaGS3* in hexaploid wheat, systematically evaluated the evolutionary progress of GS3 in plant species, and designed a functional marker for *TaGS3-7A*. Wheat carries three *TaGS3* homologs, for which the amino acid sequences contain a similar atypical Gγ domain and a cysteine-rich region that differed in length from that of rice. On the basis of sequence comparison between two extreme phenotypic pools, a KASP marker was designed and shown to have significant genetic effects on grain weight, kernel width, and kernel thickness. The present results provide novel molecular information on *GS3* and the designed functional marker may be useful in genetic improvement programs for wheat.

## Methods

### Plant materials

Wheat (*Triticum aestivum*) ‘Changzhi 6406’, which is characterized by a high grain weight in previous, was used for isolation of sequences for the three *GS3* homologs. Ten wheat accessions, divided into two pools, were used for *TaGS3-4A* and *TaGS3-7A* gene cloning; one pool comprised accessions with high grain weights (‘Changzhi 6406’, ‘Sankecun’, ‘Shannong 7859’, ‘Kangdingxiaomai’, and ‘Xingyi 4’) and the second pool consisted of accessions with low grain weight (‘Youmangbaifu’, ‘Dongnong 101’, ‘Gansu 96’, ‘Fuzhuang 30’, and ‘Bima 4’) [2].

The MCC germplasm collection, comprising 224 wheat accessions representing high genetic diversity in China, were used for validation of the association of the genetic marker and kernel-related traits (kernel length, kernel width, kernel thickness, and 1000-kernel weight) in three independent environments (E1: Luoyang at 2002; E2 Luoyang at 2005; E3 Shunyi at 2010) [2].

A panel of 238 modern wheat cultivars grown in the main wheat-production areas of China and released in the twenty-first century were used to investigate the allelic frequency and distribution of *TaGS3-7A-A*. These accessions including the 75 accessions in southwest of China and another previously characterized 163 accessions in the central plateau and north of the Yellow River [35].

### Gene isolation and analysis

*TaGS3* homologous gene sequences were isolated using a comparative genomics method. *GS3* gene sequences for rice and wheat (GenBank accession numbers DQ355996 and KF687956, respectively) were used to search for orthologs in the wheat genome using the Ensembl Plants online resource (http://plants.ensembl.org/Triticum_aestivum/Tools/Blast?db=core). Primer sets to target a specific region for each sequence were designed with Oligo 7 software and synthesized by Tsingke Biological Technology Co., Ltd. (Beijing, China).

Since *TaGS3* gene containing high GC content and repetitive sequence, Taq DNA polymerase with GC Buffer (probably lose some fidelity. Takara, RR02AG) were selected for PCR reaction. Amplification of the gene sequences by PCR was performed in a volume of 30 μl, which contained 15 μl of 2× GC buffer I, 10 μM dNTPs, 20 μmol of each primer, 1 U LA Taq™ and ~ 100 ng template DNA. All reagents were obtained from Takara Biotechnology Co., Ltd. (http://www.takara.com.cn). The reaction protocol was 94 °C for 5 min, followed by 35 cycles of 94 °C for 45 s, 58 °C for 45 s, and 72 °C for 2 min 30 s, and final extension at 72 °C for 10 min.

The PCR products were separated by electrophoresis on 2% agarose gels. All bands were cleaned and cloned into the pEASY®-T5 Zero vector (Beijing TransGen Biotech Co., Ltd.; http://www.transgen.com.cn) and transformed into DH5α competent *Escherichia coli* cells using the heat shock method. Single clones were sequenced by Tsingke Biotech Co., Ltd. Sequence alignment and assembly was performed using DNAMAN software (http://www.lynnon.com).

All sequenced clones were aligned against the wheat reference genome (IWGSC v1.0) to confirm their chromosomal locations.

### Phylogenetic analysis

The rice GS3 (*Os03g0407400*) ortholog sequence was used to search genomic databases for common higher plants (e.g., rice, maize, Arabidopsis, soybean, sorghum, and medicago) in Ensembl Plants website (http://plants.ensembl.org/Triticum_aestivum/Tools/Blast?db=core). The gene *OsDep1*, which encodes a G protein, was used as the paralogous outgroup. Alignment of the deduced amino acids sequences was performed using MUSCLE in MEGA 7.0 software [55]. A phylogenetic tree was constructed using the neighbor-joining method, Poisson model, uniform rate, and partial deletion (site coverage off 50%) as implemented in MEGA [56]. A bootstrap analysis was performed to test the robustness of the phylogenetic construction with 1000 replications.

### SNP mining and development of a gene-specific KASP marker

Single-nucleotide polymorphisms that caused a putative amino acid change and differed in frequency between the grain weight accessions were used for marker development.

For the KASP genotyping procedure, the 10 μl PCR mixture contained 100 ng template DNA, 5 μl KASP Master mix, 4 mM MgCl_2_, and 1.4 μl primer mixture, which comprised 46 μl ddH_2_O, 30 μl common primer (10 μM), and 12 μl of each tailed primer (10 μM). The PCR cycling protocol consisted of a hot start at 95 °C for 15 min, followed by ten touchdown cycles (95 °C for 20 s; touchdown at 65 °C initially and decreasing by − 1 °C per cycle for 60 s), followed by 30 additional cycles of annealing (95 °C for 10 s; 57 °C for 60 s). The extension step (each three cycles of annealing: 95 °C for 10 s; 57 °C for 60 s) was repeated three times to obtain the best genotype clustering results [35, 57]. The last 37 °C for 1 min was set up for collection of HEX and FAM fluorescence signal value. Final signal detection was applied under Bio-Rad CFX Connect™ PCR system and allele discrimination plots (clustering) generated with Bio-Rad CFX Manager™ software. The primers were synthesized by Tsingke Biological Technology Co., Ltd., and the developed KASP marker was used to screen all 224 MCC germplasm and 238 modern wheat cultivars in China.

### Statistical analysis

One-way analysis of variance was performed using IBM SPSS Statistics for Windows version 22.0 (IBM Corporation, Armonk, NY, USA) to determine the significance of differences in grain traits between the two alleles. Phenotypic data recorded in three independent environments (E1: Luoyang at 2002; E2: Luoyang at 2005; E3: Beijing at 2010) were derived from a previous study [26]. Accessions either with indistinct genotyping signal or lacking phenotype data were excluded from analyses.

## Supplementary information

**Additional file 1: Table S1.** Allelic variation of TaGS3-7A in Chinese mini-core collection based on Kompetitive allele-specific PCR (KASP) marker designed for the SNP at position 1907 (A/G). **Table S2.** Allelic variation of *TaGS3-7A* in main wheat production areas (Hebei, Shandong, Shaanxi, Henan and Sichuan) in China based on Kompetitive allele-specific PCR (KASP) marker designed for the SNP at position 1907 (A/G).

## Data Availability

All data generated or analyzed during this study are included in this published article (and additional files). The kernel traits of mini-core collection (MCC) germplasms are derived from the following public publication: Ma L, Li T, Hao C, Wang Y, Chen X, Zhang X: TaGS5-3A, a grain size gene selected during wheat improvement for larger kernel and yield. Plant Biotechnol J 2016, 14(5):1269–1280. 10.1111/pbi.12492.

## Abbreviations

ALA: Alanine
bp: Base pair
GS3: Grain shape 3
KASP: Kompetitive Allele-Specific PCR
MEGA: Molecular Evolutionary Genetic Analysis
PCR: Polymerase Chain Reaction
QTL: Quantitative trait locus
SNP: Single Nucleotide Polymorphism
THR: Threonine
UTR: Untranslated region
