# Supplementary material for: Cloning, characterization of TaGS3 and identification of allelic variation associated with kernel traits in wheat (Triticum aestivum L.)
Source: BMC Genet. 2019 Dec 18;20:98. doi: 10.1186/s12863-019-0800-6 (PMC6921503; doi:10.1186/s12863-019-0800-6)
Supplement: Supplementary file 1 — Additional file 1: Table S1. Allelic variation of TaGS3-7A in Chinese mini-core collection based on Kompetitive allele-specific PCR (KASP) marker designed for the SNP at position 1907 (A/G). Table S2. Allelic variation of TaGS3-7A in main wheat production areas (Hebei, Shandong, Shaanxi, Henan and Sichuan) in China based on Kompetitive allele-specific PCR (KASP) marker designed for the SNP at position 1907 (A/G). [file 12863_2019_800_MOESM1_ESM.docx]

**Cloning, characterization of *TaGS3* and identification of allelic variation associated with kernel traits in wheat (*Triticum* *aestivum* L.)**

Jian Yang^1^, Yanjie Zhou^1^, Yu’e Zhang^1^, Weiguo Hu^1^, Qiuhong Wu^2^, Yongxing Chen^2^, Xicheng Wang^1^, Guanghao Guo^2^, Zhiyong Liu^2*^, Tingjie Cao^1*^, Hong Zhao^1^

1 National Laboratory of Wheat Engineering, Key Laboratory of Wheat Biology and Genetic Breeding in Central Huang‑Huai Region, Ministry of Agriculture, Institute of Wheat, Henan Academy of Agricultural Sciences, Zhengzhou 450002, Henan, China

2 State Key Laboratory of Plant Cell and Chromosome Engineering, Institute of Genetics and Developmental Biology, Chinese Academy of Sciences, Beijing 100101, China

**Table S1.** Allelic variation of *TaGS3-7A* in Chinese mini-core collection based on Kompetitive allele-specific PCR (KASP) marker designed for the SNP at position 1907 (A/G).

| Accession | *TaGS3-7A* |
| --- | --- |
| Jinghong 5 | *TaGS-7A-G* |
| Neimai 11 | *TaGS-7A-G* |
| Jinmai 3 | *TaGS-7A-G* |
| Lianglaiyou | *TaGS-7A-G* |
| Bihongsui | *TaGS-7A-G* |
| Xiaobaimai | *TaGS-7A-G* |
| Hongpixiaomai | *TaGS-7A-G* |
| Xiaohongpi | *TaGS-7A-G* |
| Dingxingzhai | *TaGS-7A-G* |
| Honglidangnianlao | *TaGS-7A-G* |
| Chunxiaomai | *TaGS-7A-G* |
| Huoliangmai | *TaGS-7A-G* |
| Dahongmai | *TaGS-7A-G* |
| Shanxibaimai | *TaGS-7A-G* |
| Niuzhijia | *TaGS-7A-G* |
| Mahuaban | *TaGS-7A-G* |
| Jiahongmai | *TaGS-7A-G* |
| Hongjinmai | *TaGS-7A-G* |
| Baiqimai | *TaGS-7A-G* |
| Xiaokouhong | *TaGS-7A-G* |
| Lanhuamai | *TaGS-7A-G* |
| Zhuoludongmai | *TaGS-7A-G* |
| Laohongmai | *TaGS-7A-G* |
| Youmangbaifu | *TaGS-7A-G* |
| Hongpidongmai | *TaGS-7A-G* |
| Youmangbaifu | *TaGS-7A-G* |
| Baiqiumai | *TaGS-7A-G* |
| laomai | *TaGS-7A-A* |
| Xiaobaimai | *TaGS-7A-G* |
| Zhongyou 9507 | *TaGS-7A-A* |
| Jinmai 8 | *TaGS-7A-A* |
| Fengkang 2 | *TaGS-7A-G* |
| Changzhi 6406 | *TaGS-7A-A* |
| Beijing 8 | *TaGS-7A-G* |
| Lvzao 328 | *TaGS-7A-G* |
| Yanan 11 | *TaGS-7A-G* |
| Nongda 183 | *TaGS-7A-G* |
| Nongda 139 | *TaGS-7A-A* |
| Mingxian 169 | *TaGS-7A-G* |
| Xianmai | *TaGS-7A-G* |
| Jiangxizao | *TaGS-7A-G* |
| Honghuazao | *TaGS-7A-G* |
| Jiangdongmen | *TaGS-7A-G* |
| Dahuangpi | *TaGS-7A-G* |
| Chongyanghongmai 1 | *TaGS-7A-G* |
| Zaowutian | *TaGS-7A-G* |
| Liuzhutou | *TaGS-7A-G* |
| Chanbuzhi | *TaGS-7A-G* |
| Zhumaiyuanzitou | *TaGS-7A-G* |
| Shuilizhan | *TaGS-7A-G* |
| Huangshuibai | *TaGS-7A-G* |
| Baipu | *TaGS-7A-G* |
| Zaoxiaomai | *TaGS-7A-A* |
| Lanxizaoxiaomai | *TaGS-7A-A* |
| Wangshuibai | *TaGS-7A-A* |
| Wuyuanmai | *TaGS-7A-A* |
| Chejianzi | *TaGS-7A-G* |
| Heshangmai | *TaGS-7A-G* |
| Nuomai | *TaGS-7A-G* |
| Mangxiaomai | *TaGS-7A-G* |
| Sankecun | *TaGS-7A-G* |
| Paozimai | *TaGS-7A-G* |
| Huadong 6 | *TaGS-7A-G* |
| Liying 5 | *TaGS-7A-G* |
| Sumai 3 | *TaGS-7A-G* |
| Yangmai 158 | *TaGS-7A-G* |
| Enmai 4 | *TaGS-7A-G* |
| Emai 6 | *TaGS-7A-G* |
| Anhui 3 | *TaGS-7A-G* |
| Zhemai 1 | *TaGS-7A-G* |
| Baiyoumai | *TaGS-7A-G* |
| Yangmai | *TaGS-7A-G* |
| Dunhuachunmai | *TaGS-7A-G* |
| Huoqiu | *TaGS-7A-G* |
| Daqingmang | *TaGS-7A-G* |
| Guangtou | *TaGS-7A-G* |
| Xinkezao 9 | *TaGS-7A-G* |
| Kefeng 3 | *TaGS-7A-G* |
| Kelao 4 | *TaGS-7A-G* |
| Xinshuguang 1 | *TaGS-7A-G* |
| Dongnong 101 | *TaGS-7A-G* |
| Xinshuguang 6 | *TaGS-7A-G* |
| Jichun 1016 | *TaGS-7A-G* |
| Chixiaomai | *TaGS-7A-A* |
| Funo | *TaGS-7A-A* |
| KaBKa 3 | *TaGS-7A-A* |
| St2422/464 | *TaGS-7A-G* |
| Orofen | *TaGS-7A-G* |
| Nonglin 10 | *TaGS-7A-A* |
| Shuiyuan86 | *TaGS-7A-G* |
| Qianjiaomai | *TaGS-7A-G* |
| EarlyPremium | *TaGS-7A-G* |
| Lovrin 10 | *TaGS-7A-G* |
| Aosade 3 | *TaGS-7A-G* |
| Tanori | *TaGS-7A-G* |
| VillaGlori | *TaGS-7A-A* |
| Atlas 66 | *TaGS-7A-G* |
| Gansu 96 | *TaGS-7A-G* |
| Chaoanxiaomai | *TaGS-7A-G* |
| Chike | *TaGS-7A-G* |
| Shengen | *-* |
| Dixiuzao | *TaGS-7A-G* |
| Jingyang 60 | *TaGS-7A-G* |
| Shite 14 | *TaGS-7A-G* |
| Fuzhuang 30 | *TaGS-7A-G* |
| Bima 1 | *TaGS-7A-G* |
| Bima 4 | *TaGS-7A-G* |
| Shijiazhuang 54 | *TaGS-7A-G* |
| Pingyang 27 | *TaGS-7A-G* |
| Fengchan 3 | *-* |
| Taishan 1 | *-* |
| Jinan 2 | *-* |
| Bainong 3217 | *TaGS-7A-G* |
| Yannong 15 | *TaGS-7A-A* |
| Xinong 6028 | *TaGS-7A-G* |
| Shijiazhuang 407 | *TaGS-7A-G* |
| 12040 | *TaGS-7A-G* |
| Neixiang 5 | *TaGS-7A-G* |
| Zhengzhou 6 | *TaGS-7A-G* |
| Xiannong 39 | *TaGS-7A-G* |
| Jinan 17 | *TaGS-7A-G* |
| Xiaoyan 6 | *-* |
| Shannong 7859 | *-* |
| Aifeng 3 | *-* |
| Lumai 1 | *TaGS-7A-A* |
| Wenmai 6 | *TaGS-7A-G* |
| Laizhou 953 | *TaGS-7A-G* |
| Zhengzhou 741 | *TaGS-7A-G* |
| Baimangmai | *TaGS-7A-G* |
| Huangguaxian | *TaGS-7A-G* |
| Banjiemang | *TaGS-7A-G* |
| Laolaixia | *TaGS-7A-G* |
| Gulouding | *TaGS-7A-G* |
| Xishanbiansui | *TaGS-7A-G* |
| Honggoudou | *-* |
| Baihuomai | *-* |
| Sanyuehuang | *TaGS-7A-G* |
| Hongqiangchang | *TaGS-7A-G* |
| Youzimai | *TaGS-7A-G* |
| Pingyuan 50 | *TaGS-7A-G* |
| Baibiansui | *TaGS-7A-G* |
| Baiqimai | *TaGS-7A-G* |
| Baituzitou | *-* |
| Youmangsaogudan | *TaGS-7A-G* |
| Fuyanghong | *TaGS-7A-G* |
| Mazhamai | *TaGS-7A-G* |
| Qiangchangmang | *-* |
| Huomai | *-* |
| Meiqianhong | *TaGS-7A-G* |
| Jianmai | *TaGS-7A-G* |
| Sanyuehuang | *TaGS-7A-G* |
| Xiaofushou | *TaGS-7A-G* |
| Hongheshangtou | *TaGS-7A-G* |
| Dakoumai | *TaGS-7A-G* |
| Tumangmai | *TaGS-7A-G* |
| Baitiaoyu | *TaGS-7A-G* |
| baimangmai | *TaGS-7A-G* |
| Dayuhua | *TaGS-7A-A* |
| Fumai | *TaGS-7A-G* |
| Dalibanmang | *TaGS-7A-A* |
| Liuyuehuang | *TaGS-7A-G* |
| Gejiaxiang | *TaGS-7A-G* |
| Geerhongmai | *TaGS-7A-G* |
| Dachuanbaisilingmai | *TaGS-7A-G* |
| Bailanghuimai | *TaGS-7A-G* |
| Bendihuanghuamai | *TaGS-7A-G* |
| Motuoxiaomai | *TaGS-7A-G* |
| Bianbachunmai6 | *TaGS-7A-G* |
| Baimangxiaomai | *TaGS-7A-G* |
| Wujiangzhuo | *TaGS-7A-G* |
| Kangdingxiaomai | *-* |
| Zangdong 4 | *TaGS-7A-A* |
| Rikaze 54 | *TaGS-7A-G* |
| Rikaze 8 | *TaGS-7A-A* |
| Yizhimai | *TaGS-7A-G* |
| Dabaimai | *TaGS-7A-G* |
| Zhalaohan | *TaGS-7A-A* |
| Huoliyan | *-* |
| Shanmai | *-* |
| Hongtuzi | *-* |
| Baimazha | *-* |
| Laotutou | *-* |
| Gaoyuan 506 | *-* |
| Chunchun 28 | *-* |
| Ningchun 4 | *-* |
| Huzhuhong | *-* |
| Jinmai 4 | *TaGS-7A-G* |
| Dingxi 24 | *TaGS-7A-G* |
| Huining 10 | *TaGS-7A-A* |
| Shuwan 8 | *TaGS-7A-G* |
| Bimai 26 | *-* |
| Guinong 10 | *TaGS-7A-A* |
| Yunmai 34 | *TaGS-7A-A* |
| Xingyi 4 | *TaGS-7A-G* |
| Fengmai 11 | *TaGS-7A-G* |
| Tongjiabaxiaomai | *TaGS-7A-G* |
| Chengduguangtou | *TaGS-7A-G* |
| Baihuamai | *TaGS-7A-G* |
| Huanjiangguo | *TaGS-7A-G* |
| Hanzhongbai | *TaGS-7A-G* |
| Xiaosanyuehuang | *TaGS-7A-G* |
| Lengtiaohongmai | *-* |
| Zipi | *-* |
| Baimangmai | *-* |
| Hongmangzi | *TaGS-7A-A* |
| Yuqiumai | *TaGS-7A-G* |
| Yangmai | *TaGS-7A-A* |
| Yangmai | *TaGS-7A-G* |
| Zhushimai | *TaGS-7A-G* |
| Changmngshibiantou | *TaGS-7A-G* |
| Zhugoumai | *TaGS-7A-G* |
| Zhenxihongkeyangmai | *TaGS-7A-G* |
| Baidongmai | *TaGS-7A-G* |
| Hongchunmai | *-* |
| Chunmai | *-* |
| Hongdongmai | *TaGS-7A-A* |
| Hongdongmai | *TaGS-7A-G* |
| Wumangchunmai | *TaGS-7A-G* |
| Hongchunmai | *TaGS-7A-G* |
| Hongjinbaoyin | *TaGS-7A-G* |
| Wumangchunmai | *TaGS-7A-G* |
| Xindong 2 | *TaGS-7A-G* |
| Kashi 1 | *TaGS-7A-G* |
| Kashibaipi | *TaGS-7A-G* |

**Table S2.** Allelic variation of *TaGS3-7A* in main wheat production areas (Hebei, Shandong, Shaanxi, Henan and Sichuan) in China based on Kompetitive allele-specific PCR (KASP) marker designed for the SNP at position 1907 (A/G).

| Cultivar | Province | *TaGS3-7A* |
| --- | --- | --- |
| Cangmai 5 | Hebei | *TaGS3-7A-A* |
| Fengyou 68 | Hebei | *TaGS3-7A-A* |
| Han 6172 | Hebei | *TaGS3-7A-A* |
| Hanmai 12 | Hebei | *TaGS3-7A-A* |
| Hanmai 13 | Hebei | *TaGS3-7A-A* |
| Hanmai 14 | Hebei | *TaGS3-7A-A* |
| Hanmai 16 | Hebei | *TaGS3-7A-A* |
| Hannong 1412 | Hebei | *TaGS3-7A-A* |
| Henong 7106 | Hebei | *TaGS3-7A-A* |
| Heng 0628 | Hebei | *TaGS3-7A-G* |
| Heng 136 | Hebei | *TaGS3-7A-G* |
| Heng 4399 | Hebei | *TaGS3-7A-A* |
| Heng 4444 | Hebei | *TaGS3-7A-A* |
| Heng 6632 | Hebei | *TaGS3-7A-G* |
| Heng s29 | Hebei | *TaGS3-7A-G* |
| Hengguan 35 | Hebei | *TaGS3-7A-G* |
| Jimai 585 | Hebei | *TaGS3-7A-G* |
| Lunxuan 103 | Hebei | *TaGS3-7A-G* |
| Shijiazhuang 8 | Hebei | *TaGS3-7A-G* |
| Shimai 15 | Hebei | *TaGS3-7A-G* |
| Shimai 18 | Hebei | *TaGS3-7A-G* |
| Shimai 19 | Hebei | *TaGS3-7A-G* |
| Shimai 22 | Hebei | *TaGS3-7A-G* |
| Shinong 086 | Hebei | *TaGS3-7A-A* |
| Shiluan 02-1 | Hebei | *TaGS3-7A-A* |
| Xingmai 4 | Hebei | *TaGS3-7A-A* |
| Xingmai 6 | Hebei | *TaGS3-7A-A* |
| Xingmai 7 | Hebei | *TaGS3-7A-A* |
| Yaomai 16 | Hebei | *TaGS3-7A-A* |
| Yingbo 700 | Hebei | *TaGS3-7A-A* |
| Yongmai 1 | Hebei | *TaGS3-7A-A* |
| Zhongmai 155 | Hebei | *TaGS3-7A-A* |
| Zhongxinmai 9 | Hebei | *TaGS3-7A-A* |
| KN199 | Hebei | *TaGS3-7A-G* |
| ND399 | Hebei | *TaGS3-7A-G* |
| Lumai 15 | Shandong | *TaGS3-7A-G* |
| Lumai 16 | Shandong | *TaGS3-7A-G* |
| Lumai 17 | Shandong | *TaGS3-7A-G* |
| Lumai 21 | Shandong | *TaGS3-7A-G* |
| Lumai 22 | Shandong | *TaGS3-7A-A* |
| Lumai 23 | Shandong | *TaGS3-7A-G* |
| Luyuan 502 | Shandong | *TaGS3-7A-G* |
| Huaimai 8 | Shandong | *TaGS3-7A-A* |
| Zimai 12 | Shandong | *TaGS3-7A-A* |
| Yaonong 15 | Shandong | *TaGS3-7A-A* |
| Shannong 17 | Shandong | *TaGS3-7A-A* |
| Shannong 22 | Shandong | *TaGS3-7A-G* |
| Shannong 660 | Shandong | *TaGS3-7A-A* |
| Jinan 17 | Shandong | *TaGS3-7A-G* |
| Jinan 19 | Shandong | *TaGS3-7A-A* |
| Jimai 22 | Shandong | *TaGS3-7A-A* |
| Taishan 9818 | Shandong | *TaGS3-7A-A* |
| Liangxing 66 | Shandong | *TaGS3-7A-A* |
| Lumai 11 | Shandong | *TaGS3-7A-A* |
| Lumai 14 | Shandong | *TaGS3-7A-A* |
| Yannong 19 | Shandong | *TaGS3-7A-A* |
| Yannong 24 | Shandong | *TaGS3-7A-G* |
| Wennong 14 | Shandong | *TaGS3-7A-A* |
| Wennong 15 | Shandong | *TaGS3-7A-A* |
| Xiaoyan 6 | Shaanxi | *TaGS3-7A-A* |
| Xinong 509 | Shaanxi | *TaGS3-7A-G* |
| Xinong 511 | Shaanxi | *TaGS3-7A-A* |
| Xinonhg 529 | Shaanxi | *TaGS3-7A-A* |
| Xinong 585 | Shaanxi | *TaGS3-7A-G* |
| Xinong 109 | Shaanxi | *TaGS3-7A-G* |
| Xinong 979 | Shaanxi | *TaGS3-7A-G* |
| Xinong 529 | Shaanxi | *TaGS3-7A-A* |
| Xinong 998 | Shaanxi | *TaGS3-7A-G* |
| Xinong 18 | Shaanxi | *TaGS3-7A-G* |
| Xinong 0828 | Shaanxi | *TaGS3-7A-G* |
| Xinong 807 | Shaanxi | *TaGS3-7A-A* |
| Xinong 061 | Shaanxi | *TaGS3-7A-A* |
| Xinong 271 | Shaanxi | *TaGS3-7A-A* |
| Xiaoyan 101 | Shaanxi | *TaGS3-7A-G* |
| Xinong 533 | Shaanxi | *TaGS3-7A-A* |
| Xinong 5212 | Shaanxi | *TaGS3-7A-A* |
| Xinong 0752 | Shaanxi | *TaGS3-7A-G* |
| Xinong 188 | Shaanxi | *TaGS3-7A-A* |
| Zhengmai 379 | Henan | *TaGS3-7A-G* |
| Zhengmai 004 | Henan | *TaGS3-7A-G* |
| Jun-16 | Henan | *TaGS3-7A-G* |
| Aikang 58 | Henan | *TaGS3-7A-A* |
| Zhengmai 9023 | Henan | *TaGS3-7A-G* |
| Zhengmai 7698 | Henan | *TaGS3-7A-A* |
| Zhoumai 32 | Henan | *TaGS3-7A-G* |
| Yumai 49-198 | Henan | *TaGS3-7A-G* |
| Luomai 9 | Henan | *TaGS3-7A-G* |
| Luomai 8 | Henan | *TaGS3-7A-G* |
| Yujiao 5 | Henan | *TaGS3-7A-G* |
| Huapei 8 | Henan | *TaGS3-7A-G* |
| Huapei 6 | Henan | *TaGS3-7A-G* |
| Huapei 3 | Henan | *TaGS3-7A-A* |
| Huapei 1 | Henan | *TaGS3-7A-G* |
| Zhengyumai 9987 | Henan | *TaGS3-7A-G* |
| Zongmai 998 | Henan | *TaGS3-7A-A* |
| Suike 2 | Henan | *TaGS3-7A-A* |
| Fumai 2008 | Henan | *TaGS3-7A-A* |
| Zhengnong 17 | Henan | *TaGS3-7A-G* |
| Zhongyu 9 | Henan | *TaGS3-7A-A* |
| Zhengnong 16 | Henan | *TaGS3-7A-G* |
| Yunong 9901 | Henan | *TaGS3-7A-A* |
| Zhoumai 23 | Henan | *TaGS3-7A-A* |
| Taixue 7 | Henan | *TaGS3-7A-A* |
| Pingmai 998 | Henan | *TaGS3-7A-A* |
| Xiangmai 969 | Henan | *TaGS3-7A-A* |
| Zhongyu 10 | Henan | *TaGS3-7A-G* |
| Yunong 416 | Henan | *TaGS3-7A-G* |
| Yunong 982 | Henan | *TaGS3-7A-G* |
| Zhongyu 8 | Henan | *TaGS3-7A-G* |
| Yunong 202 | Henan | *TaGS3-7A-G* |
| Yunong 201 | Henan | *TaGS3-7A-G* |
| Zhengmai 583 | Henan | *TaGS3-7A-G* |
| Xuke 316 | Henan | *TaGS3-7A-G* |
| Xuke 1 | Henan | *TaGS3-7A-G* |
| Xuke 415 | Henan | *TaGS3-7A-G* |
| Xuke 718 | Henan | *TaGS3-7A-G* |
| Zhongyu 9398 | Henan | *TaGS3-7A-G* |
| 04zhong 36 | Henan | *TaGS3-7A-G* |
| Pumai 9 | Henan | *TaGS3-7A-G* |
| Luomai 24 | Henan | *TaGS3-7A-G* |
| Luomai 22 | Henan | *TaGS3-7A-G* |
| Luomai 21 | Henan | *TaGS3-7A-G* |
| Luomai 1 | Henan | *TaGS3-7A-G* |
| Zhongyu 6 | Henan | *TaGS3-7A-G* |
| Zhongyu 5 | Henan | *TaGS3-7A-G* |
| Zhongyu 12 | Henan | *TaGS3-7A-G* |
| Xinyuan 958 | Henan | *TaGS3-7A-G* |
| Wen 9519 | Henan | *TaGS3-7A-A* |
| Fanmai 5 | Henan | *TaGS3-7A-A* |
| Fanmai 3 | Henan | *TaGS3-7A-A* |
| Fanmai 8 | Henan | *TaGS3-7A-G* |
| Wen9629 | Henan | *TaGS3-7A-G* |
| Anmai 1 | Henan | *TaGS3-7A-G* |
| Zhoumai 16 | Henan | *TaGS3-7A-G* |
| Yubao1 | Henan | *TaGS3-7A-G* |
| Zhoumai 20 | Henan | *TaGS3-7A-A* |
| Zhoumai 22 | Henan | *TaGS3-7A-A* |
| Fengwu 981 | Henan | *TaGS3-7A-A* |
| Zhoumai 24 | Henan | *TaGS3-7A-G* |
| Zhoumai 25 | Henan | *TaGS3-7A-G* |
| Xunong 5 | Henan | *TaGS3-7A-G* |
| Pingan 6 | Henan | *TaGS3-7A-G* |
| Pingan 8 | Henan | *TaGS3-7A-G* |
| Yuzhan 4 | Henan | *TaGS3-7A-G* |
| Xi nmai 11 | Henan | *TaGS3-7A-A* |
| Xi nmai 12 | Henan | *TaGS3-7A-G* |
| Xinmai 13 | Henan | *TaGS3-7A-A* |
| Ruzhou 0319 | Henan | *TaGS3-7A-G* |
| Xinmai 21 | Henan | *TaGS3-7A-G* |
| Xinmai 9 | Henan | *TaGS3-7A-G* |
| Zhengmai 0856 | Henan | *TaGS3-7A-G* |
| Zhengmai 9694 | Henan | *TaGS3-7A-G* |
| Xinmai16 | Henan | *TaGS3-7A-G* |
| Xinmai19 | Henan | *TaGS3-7A-G* |
| Xinmai18 | Henan | *TaGS3-7A-G* |
| Yanzhan 4110 | Henan | *TaGS3-7A-G* |
| Zhengmai 366 | Henan | *TaGS3-7A-A* |
| Zhoumai 18 | Henan | *TaGS3-7A-A* |
| Tianhe 3 | Henan | *TaGS3-7A-G* |
| Lankao 198 | Henan | *TaGS3-7A-G* |
| Kaimai 18 | Henan | *TaGS3-7A-G* |
| Kaimai 20 | Henan | *TaGS3-7A-A* |
| Kaimai 21 | Henan | *TaGS3-7A-A* |
| Chuanmai 35 | Sichuan | *TaGS3-7A-A* |
| Chuanmai 36 | Sichuan | *TaGS3-7A-G* |
| Chuanmai 38 | Sichuan | *TaGS3-7A-A* |
| Chuanmai 44 | Sichuan | *TaGS3-7A-A* |
| Chuanmai 45 | Sichuan | *TaGS3-7A-A* |
| Chaunmai 46 | Sichuan | *TaGS3-7A-A* |
| Chuanmai 47 | Sichuan | *TaGS3-7A-A* |
| Chuanmai 48 | Sichuan | *TaGS3-7A-A* |
| Chuanmai 49 | Sichuan | *TaGS3-7A-A* |
| Chuanmai 50 | Sichuan | *TaGS3-7A-A* |
| Chuanmai 51 | Sichuan | *TaGS3-7A-A* |
| Chuanmai 52 | Sichuan | *TaGS3-7A-A* |
| Chuanmai 53 | Sichuan | *TaGS3-7A-A* |
| Chuanmai 54 | Sichuan | *TaGS3-7A-A* |
| Chuanmai 56 | Sichuan | *TaGS3-7A-A* |
| Chuanmai 58 | Sichuan | *TaGS3-7A-G* |
| Chuanmai 60 | Sichuan | *TaGS3-7A-A* |
| Chuanmai 107 | Sichuan | *TaGS3-7A-A* |
| Chaunmai 104 | Sichuan | *TaGS3-7A-A* |
| Chuanchongzu 125 | Sichuan | *TaGS3-7A-A* |
| Chuannong 11 | Sichuan | *TaGS3-7A-A* |
| Chuannong 12 | Sichuan | *TaGS3-7A-A* |
| Chuannong 16 | Sichuan | *TaGS3-7A-A* |
| Chuannong 17 | Sichuan | *TaGS3-7A-A* |
| Chuannong 18 | Sichuan | *TaGS3-7A-A* |
| Chuannong 20 | Sichuan | *TaGS3-7A-A* |
| Chuannong 21 | Sichuan | *TaGS3-7A-A* |
| Chuannong 22 | Sichuan | *TaGS3-7A-G* |
| Chuannong 23 | Sichuan | *TaGS3-7A-A* |
| Chuannong 24 | Sichuan | *TaGS3-7A-A* |
| Chuannong 25 | Sichuan | *TaGS3-7A-A* |
| Chuannong 26 | Sichuan | *TaGS3-7A-A* |
| Chuannong 27 | Sichuan | *TaGS3-7A-A* |
| Mianyang 30 | Sichuan | *TaGS3-7A-A* |
| Mianyang 32 | Sichuan | *TaGS3-7A-A* |
| Mianyang 33 | Sichuan | *TaGS3-7A-A* |
| Mianyang 35 | Sichuan | *TaGS3-7A-A* |
| Mianyang 37 | Sichuan | *TaGS3-7A-A* |
| Mianyang 38 | Sichuan | *TaGS3-7A-A* |
| Mianyang 39 | Sichuan | *TaGS3-7A-A* |
| Mianyang 42 | Sichuan | *TaGS3-7A-A* |
| Mianyang 43 | Sichuan | *TaGS3-7A-A* |
| Mianyang 46 | Sichuan | *TaGS3-7A-A* |
| Mianzamai 168 | Sichuan | *TaGS3-7A-A* |
| Mianmai 185 | Sichuan | *TaGS3-7A-A* |
| Mianmai 1403 | Sichuan | *TaGS3-7A-A* |
| Chuanyu 16 | Sichuan | *TaGS3-7A-A* |
| Chuanyu 17 | Sichuan | *TaGS3-7A-A* |
| Chuanyu 18 | Sichuan | *TaGS3-7A-A* |
| Chuanyu 19 | Sichuan | *TaGS3-7A-A* |
| Chuanyu 20 | Sichuan | *TaGS3-7A-A* |
| Chuanyu 23 | Sichuan | *TaGS3-7A-A* |
| Chuanyu 24 | Sichuan | *TaGS3-7A-G* |
| Xikemai 1 | Sichuan | *TaGS3-7A-A* |
| Xikemai 2 | Sichuan | *TaGS3-7A-A* |
| Xikemai 3 | Sichuan | *TaGS3-7A-G* |
| Xikemai 4 | Sichuan | *TaGS3-7A-A* |
| Xikemai 5 | Sichuan | *TaGS3-7A-A* |
| Xikemai 6 | Sichuan | *TaGS3-7A-A* |
| Rongmai 2 | Sichuan | *TaGS3-7A-A* |
| Rongmai 3 | Sichuan | *TaGS3-7A-A* |
| Rongmai 4 | Sichuan | *TaGS3-7A-A* |
| Kechengmai 2 | Sichuan | *TaGS3-7A-A* |
| Neimai 8 | Sichuan | *TaGS3-7A-A* |
| Neimai 9 | Sichuan | *TaGS3-7A-A* |
| Neimai 11 | Sichuan | *TaGS3-7A-A* |
| Liangmai 2 | Sichuan | *TaGS3-7A-A* |
| Liangmai 3 | Sichuan | *TaGS3-7A-A* |
| Liangmai 4 | Sichuan | *TaGS3-7A-A* |
| Shumai 375 | Sichuan | *TaGS3-7A-G* |
| Xingmai 2 | Sichuan | *TaGS3-7A-A* |
| Rongmai 757 | Sichuan | *TaGS3-7A-A* |
| Chengdianmai 1 | Sichuan | *TaGS3-7A-A* |
| Yimai 8 | Sichuan | *TaGS3-7A-A* |
| Jinfeng 626 | Sichuan | *TaGS3-7A-A* |
